# Screening and follow‐up of chronic liver diseases with understanding their etiology in clinics and hospitals

**DOI:** 10.1002/jgh3.12406

**Published:** 2020-08-24

**Authors:** Masahiro Ogawa, Atsunori Tsuchiya, Takayuki Watanabe, Toru Setsu, Naruhiro Kimura, Masato Matsuda, Yoshiki Hoshiyama, Hiroaki Saito, Tsutomu Kanazawa, Motoi Shiotani, Tatsuhiko Sato, Takuya Yagi, Koji Igarashi, Norihiko Yoshimura, Masaaki Takamura, Hidefumi Aoyama, Shuji Terai

**Affiliations:** ^1^ Division of Gastroenterology and Hepatology, Graduate School of Medical and Dental Sciences Niigata University Niigata Japan; ^2^ Medical Laboratory Division Niigata University Medical and Dental Hospital Niigata Japan; ^3^ Division of Radiology, Department of Clinical Technology Niigata University Medical and Dental Hospital Niigata Japan; ^4^ Department of Radiology and Radiation Oncology Niigata University Graduate School of Medical and Dental Sciences Niigata Japan; ^5^ Bioscience Division TOSOH Corporation Ayase‐shi Japan

**Keywords:** autotaxin, fibrosis‐4 index, hepatocellular carcinoma, Mac‐2‐binding protein glycosylation isomer, magnetic resonance elastography

## Abstract

**Background and Aim:**

Considering the increasing prevalence of non‐alcoholic fatty liver disease and non‐alcoholic steatohepatitis (NASH), the development of an effective screening and follow‐up system that enables the recognition of etiological changes by primary physicians in clinics and specialists in hospitals is required.

**Methods:**

Chronic hepatitis B (HBV) and C (HCV), NASH, and alcoholic steatohepatitis (ASH) patients who were assayed for Mac‐2‐binding protein glycosylation isomer (M2BPGi) (*n* = 272) and underwent magnetic resonance elastography (MRE) (*n* = 119) were enrolled. Patients who underwent MRE were also tested by ultrasound elastography (USE) (*n* = 80) and for M2BPGi (*n* = 97), autotaxin (ATX) (*n* = 62), and platelet count (*n* = 119), and their fibrosis‐4 (FIB‐4) index was calculated (*n* = 119).

**Results:**

FIB‐4 index >2, excluding HBV‐infected patients, M2BPGi >0.5, ATX >0.5, and platelet count <20 × 10^4^/μL were the benchmark indices, and we took into consideration other risk factors, such as diabetes mellitus and age, to recommend further examinations, such as USE, based on the local situation to avoid overlooking hepatocellular carcinoma (HCC) in the clinic. During specialty care in the hospital, MRE exhibited high diagnostic ability for fibrosis stages >F3 or F4; it could efficiently predict collateral circulation with high sensitivity, which can replace USE. We also identified etiological features and found that collateral circulation in NASH/ASH patients tended to exceed high‐risk levels; moreover, these patients exhibited more variation in HCC‐associated liver stiffness than the HBV and HCV patients.

**Conclusions:**

Using appropriate markers and tools, we can establish a stepwise, practical, noninvasive, and etiology‐based screening and follow‐up system in primary and specialty care.

## Graphical abstract

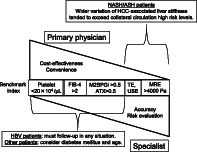

## Introduction

A recently developed treatment for hepatitis C that uses direct‐acting antivirals (DAAs) has achieved nearly 100% sustained virologic response (SVR).^1^, ^2^ In the case of hepatitis B, nucleoside analog therapies can control the infection, whereas vaccination and reducing vertical transmission can decrease the number of patients.^3^ Therefore, the etiology of liver cirrhosis is now shifting from a primarily virus‐related hepatitis disease to predominantly alcoholic and non‐alcoholic steatohepatitis (ASH and NASH, respectively).^4^ Patients with ASH or NASH tend to be misdiagnosed and are not followed up, unlike patients with hepatitis B and C, who are regularly followed up. The diagnosis of hepatocellular carcinoma (HCC) in these patients is often delayed, and at the time of detection, the HCCs are often at an advanced stage.^5^, ^6^ Thus, the development of an effective screening and follow‐up system to detect this etiological change in the clinic by primary physicians and in the hospital by specialists is critical.

Recent large‐scale cohort studies showed that fibrosis determines the outcomes in non‐alcoholic fatty liver disease (NAFLD),^7^, ^8^, ^9^ and in other types of chronic liver diseases, evaluating fibrosis is one of the most important steps for the follow‐up of patients in the clinic and hospital.^10^, ^11^ In addition, the course of disease progression and the intervention or treatment strategy differ based on the etiology of chronic liver diseases. Clinics and hospitals play specific roles in this process. Clinics need to quickly and cost‐effectively diagnose high‐risk diseases; in contrast, hospital specialists conduct a detailed and accurate follow‐up of patients.

The most critical, game‐changing intervention for the HCC course remains the improvement of detection rates at an early stage. To achieve this goal, the most useful approach may be to optimize screening strategies to better identify higher‐risk patients who require a more intense survey.^12^ Our knowledge of the cost‐effectiveness of HCC surveillance is based on model‐based studies because following up with patients without performing surveillance is not an option for trials as surveillance has become the standard of care globally. HCC surveillance with ultrasound elastography (USE) ± α‐fetoprotein (AFP) does not constitute direct physical harm; however, frequently observed false negative results require subsequent computed tomography and/or magnetic resonance imaging (MRI) at shorter intervals, which increases radiation exposure, possible contrast injury, and financial burden. The key to increasing the yield and cost‐effectiveness lies in a risk‐stratified surveillance strategy.^13^

In this study, we first focused on serum markers that had recently become available, namely, *Wisteria floribunda* agglutinin+—Mac‐2 binding protein (WFA+‐M2BP; M2BPGi),^14^, ^15^ autotaxin (ATX),^16^ platelet count,^17^ and the fibrosis‐4 (FIB‐4) index,^18^ to generate data that took into consideration HCC patients in the clinic. Next, we analyzed the elastography data using ultrasound (US)^19^, ^20^ and MRI^21^, ^22^, ^23^, ^24^, ^25^ to efficiently predict the following: collateral circulation (with high sensitivity), characteristics associated with liver stiffness and liver function, and HCC occurrence depending on the etiology. Using this method, we tried to enable stepwise, practical, noninvasive, and etiology‐based screening and follow‐up at the levels of primary and specialty care based on the status of a given facility.

## Methods

### *Patients and specimens*

Inpatients and outpatients at the Niigata University Hospital, from 2016 to 2018, who were tested for M2BPGi (total, *n* = 272; hepatitis B virus [HBV], *n* = 82; hepatitis C virus [HCV], *n* = 88; NASH, *n* = 45; ASH, *n* = 59) and ATX (total, *n* = 122; HBV, *n* = 25; HCV, *n* = 25; NASH, *n* = 26; ASH, *n* = 15; others, *n* = 31) and underwent magnetic resonance elastography (MRE) (total, *n* = 119; HBV, *n* = 38; HCV, *n* = 28; NASH, *n* = 19; ASH, *n* = 15; others, *n* = 19) were enrolled. The patients who underwent MRE were also subjected to USE, using acoustic radiation force impulse (ARFI) (*n* = 80), and assayed for M2BPGi (*n* = 97), ATX (*n* = 62), and platelet count (*n* = 119); in addition, their FIB‐4 index was also calculated (*n* = 119). For studies on noninvasive markers for HCC detection, the data for M2BPGi (*n* = 272), ATX (*n* = 122), platelet count (*n* = 119), and FIB‐4 index (*n* = 119) were used. For studies on the correlation of the other parameters with MRE, the data for MRE (*n* = 119) and their corresponding platelet count (*n* = 119), FIB‐4 (*n* = 119), ATX (*n* = 62), M2BPGi (*n* = 97), and USE (*n* = 80) data were used. For studies on the correlation of MRE with USE for specialist use, MRE (*n* = 119) and USE (*n* = 80) data were used (Table S1 and Figure S1, Supporting information). Patients who underwent MRE and surgery for HCC (*n* = 23) or echo‐guided liver biopsy (*n* = 8) using a fine biopsy needle (Majima needle) for the diagnosis of the etiology of the liver disease were enrolled in the study to verify the relationship between liver stiffness, as calculated by MRE, and the fibrosis stage of the liver tissue. This retrospective study was approved by the Institutional Review Board (IRB) of Niigata University. Written informed consent was obtained from each patient, and the study protocol conformed to the ethical guidelines of the 1975 Declaration of Helsinki.

A further description of the materials and methods used is provided in the supplemental information.

## Results

### *The role of noninvasive markers in the detection of* *HCC*

**Figure 1:**
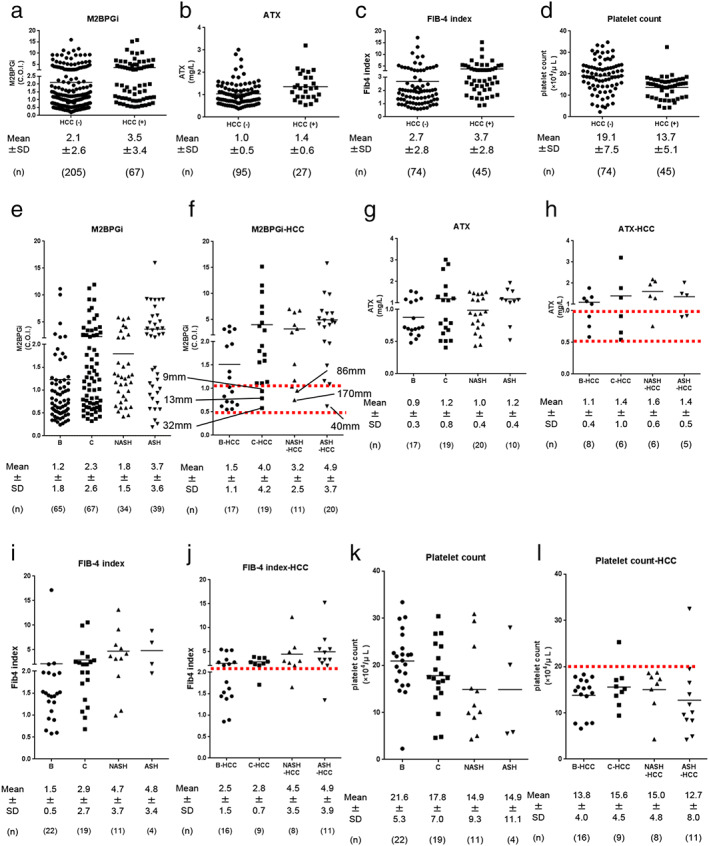
Relatively convenient fibrosis markers for primary physicians. M2BPGi (a), ATX (b), FIB‐4 index (b), and platelet count (d) with or without hepatocellular carcinoma (HCC). M2BPGi (e, f), ATX (g, h), FIB‐4 index (i, j), and platelet count (k, l) without (e, g, i, k) or with HCC (f, h, j, l) depending on etiology. Red dotted lines in (f) indicate the 0.5 cut‐off index (COI) and 1.0 COI; in (h), 0.5 mg/L and 1.0 mg/L; in (j), 2 FIB‐4 index; and in (l), 20 × 10^4^/μL on the *y* axis.

To evaluate which markers are effective for identifying HCC patients in the clinic, we assessed common fibrosis markers, such as M2BPGi, ATX, FIB‐4 index, and platelet count (without distinguishing them based on the etiology [HBV, HCV, NASH, and ASH]), in patients with or without HCC. As shown in Figure 1, these markers were unable to distinguish between the patients with or without HCC when analyzed without considering the etiology (Fig. 1a–d). Next, we evaluated the M2BPGi, ATX levels, FIB‐4 index, and platelet count of the patients with or without HCC, depending on the etiology (Fig. 1e–L). In each case, the levels of the following markers were lower in the HBV patients with HCC: M2BPGi (HBV, HCV, NASH: and ASH; 1.5 ± 1.1, 4.0 ± 4.2, 3.2 ± 2.5, and 4.9 ± 3.7 cut‐off index [COI], respectively), ATX (1.1 ± 0.4, 1.4 ± 1.0, 1.6 ± 0.6, and 1.4 ± 0.5 mg/L, respectively), and FIB‐4 index (2.5 ± 1.5, 2.8 ± 0.7, 4.5 ± 3.5, and 4.9 ± 3.9, respectively), but the platelet count was almost the same in these patients (13.8 ± 4.0, 15.6 ± 4.5, 15.0 ± 4.8, 12.7 ± 8.0 × 10^4^/μL, respectively), suggesting that HBV patients must be followed up regardless of the levels of these fibrosis markers. For all other etiologies, HCC was found in all patients with FIB‐4 index >2 (sensitivity 75.6%; specificity 60.8%), except for three patients (one patient each for HCV, NASH, and ASH). Therefore, we propose that an FIB‐4 index >2 might be sufficient to predict HCC. With regard to the other markers, platelet count <20 × 10^4^/μL (sensitivity 95.5%; specificity 44.6%), M2BPGi >1 (sensitivity 79.1%; specificity 45.9%), and ATX >1 (sensitivity 64.0%; specificity 52.3%) might be used in addition to regular follow‐up with ultrasonography and/or tumor markers. In addition, the frequency of HCC patients presenting with 1 > M2BPGi > 0.5 or 1 > ATX > 0.5 was very low; however, this range must be considered by keeping in mind the differences in the etiologies. In HCV patients, a small‐sized HCC was detected in patients with 1 > M2BPGi > 0.5 or 1 > ATX > 0.5 (9 mm, 12 mm, and 32 mm for M2BPGi and 20 mm, 32 mm, 20 mm, and 14 mm for ATX) during regular follow‐up. In NASH and ASH patients with 1 > M2BPGi > 0.5 or 1 > ATX > 0.5, the HCCs were not followed up regularly, and the detected tumors were of a larger size (86 mm, 170 mm, 40 mm for M2BPGi and 32 mm, 75 mm, and 113 mm for ATX). NASH and ASH HCC patients with 1 > M2BPGi > 0.5 or 1 > ATX > 0.5 were characterized by diabetes mellitus (DM) and/or relatively old age (>75 years old).

### *Diagnostic ability of* *MRE,* *US, and noninvasive markers for fibrosis stages* *F3* *and* *F4*

**Figure 2:**
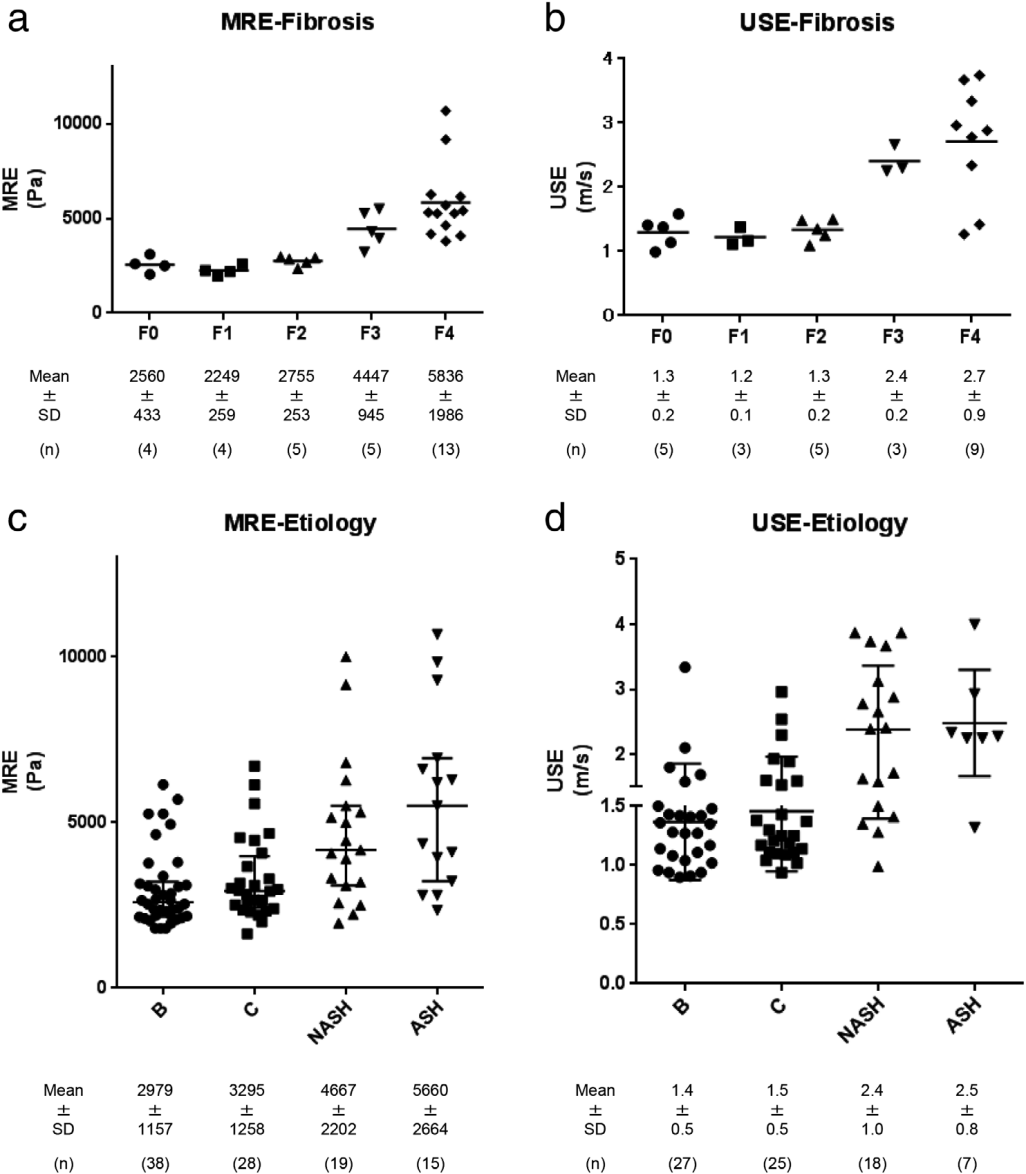
Correlation between magnetic resonance elastography (MRE) and ultrasound elastography (USE) data and between tissue fibrosis stages and MRE data of each etiology. MRE (a) and USE (b) data show that liver stiffness increased with increasing fibrosis stage. Liver stiffness measured by MRE (c) and USE (d) categorized by each etiology.

Specialists in hospitals perform detailed follow‐up to manage the risk of HCC and collateral circulation. Here, we evaluated the validity of MRE in effectively evaluating advanced fibrosis in patients. First, we compared the relationship between the available tissue fibrosis stages and MRE data and found that MRE levels increased with increasing fibrosis stages (F0; 2560 ± 433 Pa, F1; 2249 ± 259 Pa, F2; 2755 ± 253 Pa, F3; 4447 ± 945 Pa, and F4; 5836 ± 1986 Pa) (Fig. 2a). Next, we evaluated the MRE data based on the etiology and observed that patients with NASH (4667 ± 2202 Pa) and ASH (5660 ± 2664 Pa) tended to have greater liver stiffness and exhibited higher liver stiffness than patients with HBV (2979 ± 1157 Pa) and HCV (3295 ± 1258 Pa) (Fig. 2c). This tendency could also be detected using USE (Fig. 2b,d), although MRE could clearly distinguish the differences in characteristics between HBV and HCV and between NASH and ASH.

**Figure 3:**
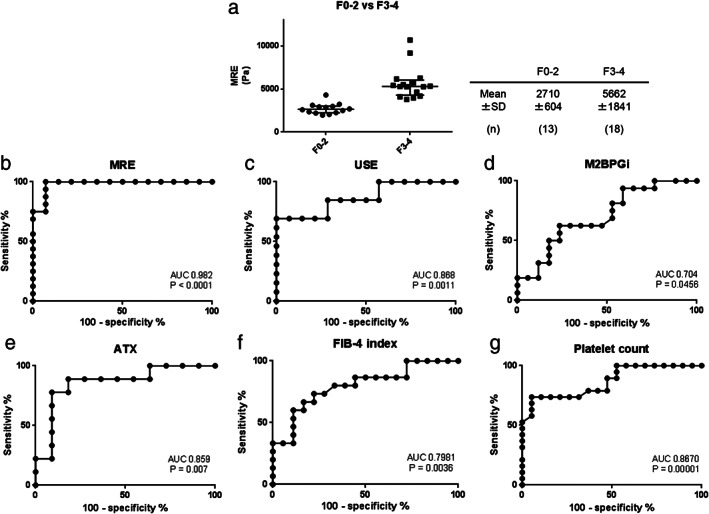
Evaluation of the ability of magnetic resonance elastography (MRE) in high fibrosis stage patients. Liver stiffness of F0–2 and F3–4 (a). AUROC for predicting fibrosis states higher than F3 by MRE (b), ultrasound elastography (USE) (c), M2BPGi (d), ATX (e), FIB‐4 index (f), and platelet count (g).

**Figure 4:**
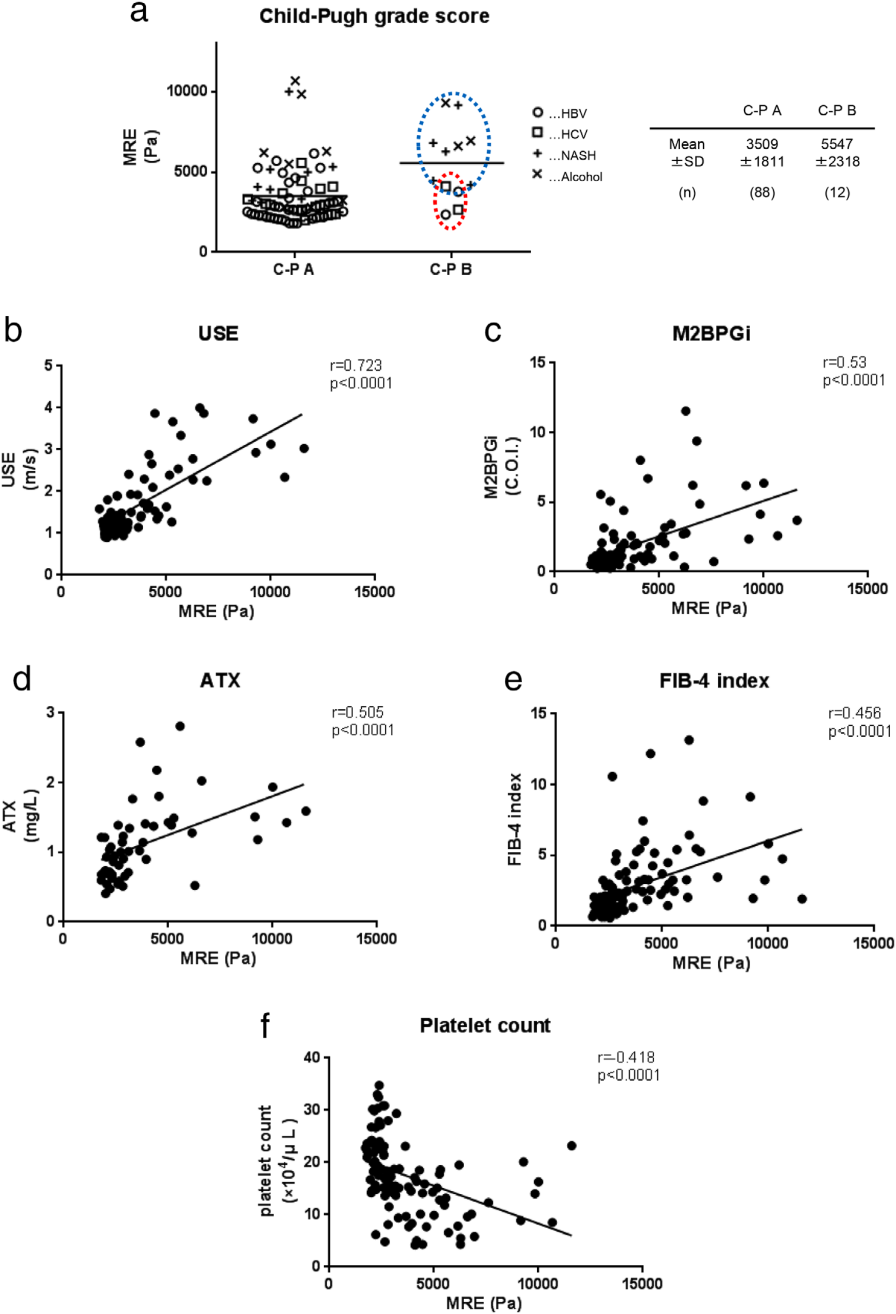
Liver stiffness and Child‐Pugh classification and correlation between magnetic resonance elastography (MRE) and each tested parameter. Liver stiffness in Child‐Pugh grade B patients differs based on etiology (a). Blue dotted circles represent non‐alcoholic steatohepatitis and alcoholic steatohepatitis patients, and red dotted circles represent hepatitis B virus and hepatitis C virus patients. Correlation between MRE and ultrasound elastography (USE) (b), M2BPGi (c), ATX (d), FIB‐4 index (e), and platelet count (f).

To further investigate the validity of MRE for the evaluation of fibrosis in patients, patients with fibrosis stages higher than F3 and F4 were analyzed. The mean F0‐2 and F3‐4 MRE levels were 2710 ± 604 Pa and 5662 ± 1841 Pa, respectively (Fig. 3a), and the Area Under the Receiver Operating Characteristics (AUROC) for predicting fibrosis stages higher than F3 was 0.982 (*P* < 0.0001), which was much higher than that of other parameters, such as USE (AUROC = 0.868, *P* = 0.0011), M2BPGi (AUROC = 0.704, *P* = 0.0456), ATX (AUROC = 0.859, *P* = 0.0007), FIB‐4 index (AUROC = 0.798, *P* = 0.0036), and platelet count (AUROC = 0.8670, *P* = 0.00001) (Fig. 3b–g). These results showed that MRE had high diagnostic potential for advanced fibrosis in patients. Next, the patients were divided into two groups, Child‐Pugh (C‐P) grade A group and grade B group, and the markers were analyzed based on etiologies. We noticed that, among C‐P grade B patients, liver stiffness was significantly higher in the NASH and ASH patients (5547 ± 2318 Pa) than in the HBV and HCV patients (3509 ± 1811 Pa) (Fig. 4a).

Finally, the analysis of the relationship between MRE and USE, M2BPGi, ATX, FIB‐4 index, and platelet count revealed that the correlation with MRE was *r* = 0.723 (*P* < 0.0001) for USE, *r* = 0.530 (*P* < 0.0001) for M2BPGi, *r* = 0.505 (*P* < 0.0001) for ATX, *r* = 0.456 (*P* < 0.0001) for FIB‐4 index, and *r* = −0.418 (*P* < 0.0001) for platelet count. These results indicated a high correlation between MRE and USE (Fig. 4b–f).

### *Efficacy of* *MR* *and* *US* *elastography in characterizing collateral circulation*

**Figure 5:**
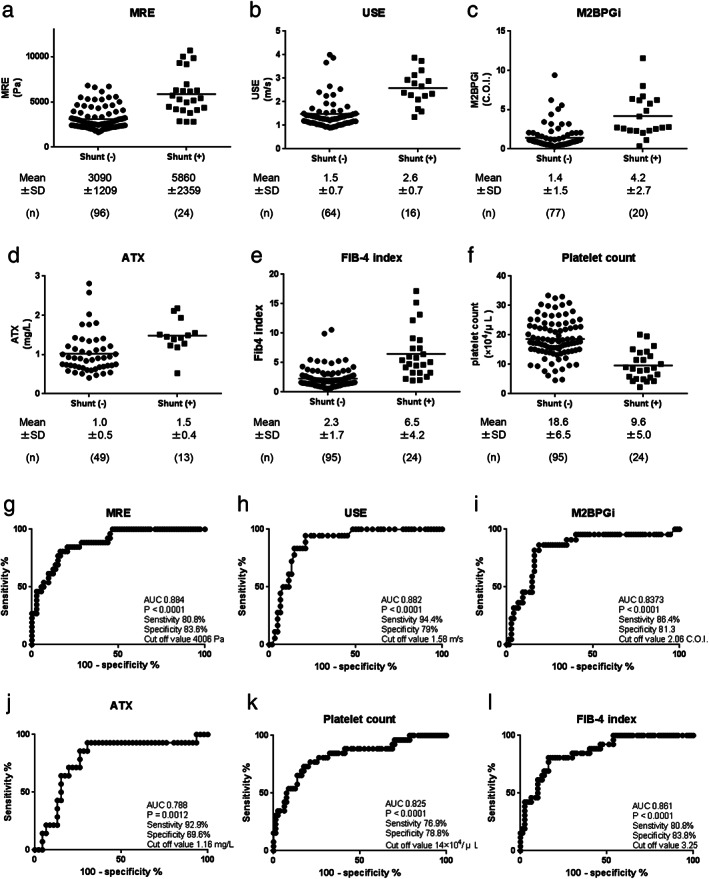
Relationship between clinical parameters and collateral circulation. Data for clinical parameters with or without collateral circulation are shown as follows: magnetic resonance elastography (MRE) (a), ultrasound elastography (USE) (b), M2BPGi (c), ATX (d), FIB‐4 index (e), and platelet count (f). AUROC values for predicting collateral circulation were analyzed as follows: MRE (g), USE (h), M2BPGi (i), ATX (j), FIB‐4 index (k), and platelet count (l).

Next, we evaluated the ability of MRE, USE, M2BPGi, ATX, FIB‐4 index, and platelet count to predict collateral circulation using AUROC analyses and found that the AUROC values were 0.884, *P* < 0.0001 (MRE); 0.882, *P* < 0.0001 (USE); 0.837, *P* < 0.0001 (M2BPGi); 0.788, *P* = 0.0012 (ATX); 0.825, *P* < 0.0001 (FIB‐4 index); and 0.861, *P* < 0.0001 (platelet count) (Fig. 5a–f). Although MRE exhibited the strongest predictive power for collateral circulation, these results revealed that each marker could accurately predict collateral circulation. When we set the cut‐off at 4006 Pa in MRE, the sensitivity and specificity for the prediction of collateral circulation were 80.6 and 83.6%, respectively. When the cut‐off was 1.58 m/s in USE, the sensitivity and specificity were 94.4 and 79.0%, respectively (Fig. 5g–l).

### *Efficacy of* *MR* *and* *US* *elastography in the identification of* *HCC‐associated liver fibrosis*

**Figure 6:**
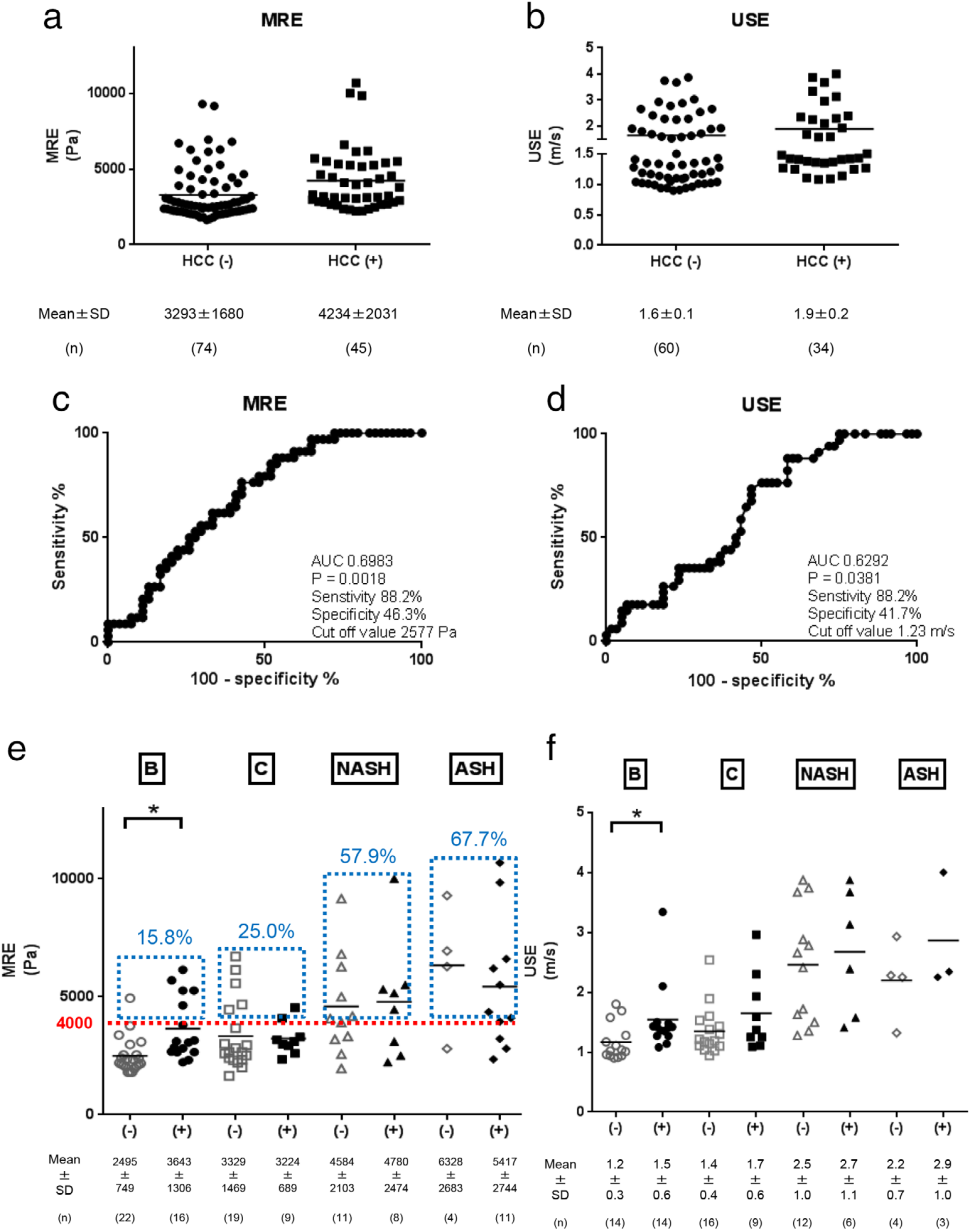
Magnetic resonance elastography (MRE) and ultrasound elastography (USE) data with and without hepatocellular carcinoma (HCC). MRE (a) and USE (b) data with and without HCC are shown. AUROC values of MRE (c) and USE (d) for predicting HCC are shown. MRE (e) and USE (f) data with and without HCC categorized by etiologies. Red dotted lines indicate liver stiffness level of 4000 Pa. The percentages indicated in blue are the frequency of patients with MRE value above 4000 Pa (indicated by red dotted line) in each etiology.

The ability of MRE and USE to predict HCC was analyzed, and the AUROC values were 0.6998, *P* = 0.0018 and 0.6292, *P* = 0.0381, respectively (Fig. 6a–d). Next, we analyzed liver stiffness in patients with HCC depending on the etiology. The mean levels of liver stiffness in the HBV, HCV, NASH, and ASH patients with HCC were 3643 ± 1306 Pa, 3224 ± 689 Pa, 4780 ± 2474 Pa, and 5417 ± 2744 Pa, respectively. Thus, the NASH and ASH patients tended to have a wider range of HCC‐associated liver stiffness compared to the HBV and HCV patients (Fig. 6e). These tendencies were also observed in the results of USE; the mean levels of liver stiffness in the HBV, HCV, NASH, and ASH patients with HCC were 1.5 ± 0.6 m/s, 1.7 ± 0.6 m/s, 2.7 ± 1.1 m/s, and 2.9 ± 1.0 m/s, respectively (Fig. 6f). These results revealed that specialists must consider HCC risk in NASH and ASH patients with low to high liver stiffness compared with HBV and HCV patients. Furthermore, the frequencies of patients with liver stiffness of >4000 Pa in MRE were as follows: HBV (15.8%), HCV (25.0%), NASH (57.9%), and ASH (67.7%) (Fig. 6e). These results suggested that, in NASH and ASH patients, the frequency of patients with collateral circulation could be higher than that in HBV and HCV patients; moreover, in these NASH and ASH patients, specialists must pay special attention to collateral circulation and HCC while considering the liver stiffness.

## Discussion

**Figure 7:**
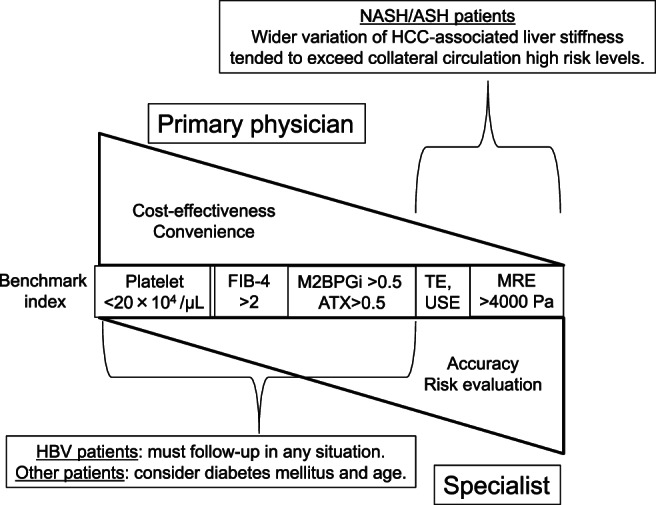
Highlight of this study. ASH, alcoholic steatohepatitis; HBV, hepatitis B virus; HCC, hepatocellular carcinoma; MRE, magnetic resonance elastography; NASH, non‐alcoholic steatohepatitis.

During screening and follow‐up, we found chronic liver disease etiology to be a critical issue for both primary clinic physicians and hospital specialists. This is particularly important as the etiology of chronic liver disease and HCC is shifting from being primarily HBV and HCV to NASH and ASH. Our results suggest that HBV patients must be regularly followed up (depending on the situation), whereas for other patients, M2BPGi >0.5, ATX >0.5, and FIB‐4 index >2 may be important screening benchmark indices for recommending further examinations, such as US, depending on the local conditions. Furthermore, depending on a patient's HCC risk, tumor marker calculation may be necessary. Patients with 1 > M2BPGi > 0.5 and 1 > ATX > 0.5 had a relatively lower risk of HCC compared to those with M2BPGi >1 and ATX >1. However, when patients of older age (>75 years) and with DM were included in the analysis, we found that patients with 1 > M2BPGi > 0.5 and 1 > ATX > 0.5 might require more attention with regard to HCC risk. The MRE and USE data showed that the liver stiffness associated with HCC varied depending on the etiology. NASH/ASH patients tended to have a wide variation in HCC‐associated liver stiffness. USE or MRE (depending on the status of the hospital) helped predict collateral circulation and HCC occurrence. MRE (>4000 Pa) could efficiently predict collateral circulation with high sensitivity, and patients with NASH/ASH tended to exceed high‐risk collateral circulation levels (Fig. 7).

ATX is a secreted lysophospholipase D that catalyzes the hydrolysis of lysophosphatidylcholine (LPC) to lysophosphatidic acid (LPA), a pleiotropic growth factor‐like phospholipid, and has been reported to promote liver fibrosis and cancer.^16^ M2BPGi, which is mainly produced by hepatic stellate cells and may interact with Mac‐2 positive cells such as macrophages to induce biological activity, is a reliable marker for assessing liver fibrosis in patients with viral hepatitis and other fibrotic liver diseases, such as primary biliary cholangitis, biliary atresia, autoimmune hepatitis, and NAFLD.^14^, ^15^ In the clinic, these markers can be useful as their levels can directly indicate risk without the need for any calculation. Although we did not assess their usefulness in the clinic, our findings indicate that these markers may predict HCC more accurately when combined with data on the age and DM condition of patients in the clinic.

Age and DM seem to be very important factors in the context of carcinogenesis in NAFLD patients.^26^ Younossi *et al*. reported that the prevalence of NAFLD and NASH in patients with type 2 (T2) DM is over 60%, and the presence of T2DM seems to accelerate the course of NAFLD and is a predictor of advanced fibrosis and mortality.^27^ We previously analyzed carcinogenesis in NASH patients and found that 58.3% of the patients had T2DM and that the mean age of HCC occurrence was 75.1 ± 5.1 years, highlighting the importance of T2DM and age.^28^ In this study, we showed that the FIB‐4 index calculated using an important factor, age, can effectively predict HCC occurrence. Ito *et al*. reported that, when patients were divided based on the FIB‐4 index into <2.67 and ≥ 2.67 groups, the FIB‐4 index ≥2.67 group displayed significantly lower overall survival rates and higher incidence of HCC and LC‐related complications than the FIB‐4 index <2.67 group.^18^ A recent study found that the optimal FIB‐4 cut‐off value in NASH patients for the identification of advanced fibrosis was >1.3 in the literature.^29^ However, for identifying HCC risk, we showed that a cut‐off value of 2 would be more appropriate.

In this report, we show that considering the etiology is very important for monitoring HCC. In patients rigorously treated for viral hepatitis, HCC tended to occur before collateral vein formation (before 4000 Pa); however, in NASH and ASH patients, HCC can occur at a very wide range of liver stiffness. Thus, in these patients, it is better to carefully follow both the HCC occurrence and collateral vein formation at the same time. USE for HCC screening is an easy approach in chronic liver disease patients. In this study, we used ARFI; however, FibroScan and Supersonic Shear Imaging are also widely used.^30^, ^31^ We believe that MRE is the most accurate method; in this study, USE showed the highest correlation with MRE among the tested markers. It has been widely reported that MRE can predict advanced fibrosis in NAFLD patients and is more accurate than transient elastography. Matsui *et al*. reported that patients with NAFLD who meet the MRE–liver stiffness measurement (LSM) criterion (<4.2 kPa) and the platelet count criterion (>18 × 10^4^/μL) do not need to undergo screening esophagogastroduodenoscopy; however, these criteria should be rechecked annually. If the LSM increases or the platelet count decreases, a screening EGD should be performed.^22^ Our study revealed that, with a liver stiffness of 4000 Pa, patients tended to have collateral circulation, which is in agreement with the criteria suggested by Matsui *et al*.

As clinical evidence has revealed that liver function improves after balloon‐occluded retrograde transvenous obliteration, the prevention of a portosystemic shunt may be very important for liver regeneration and fibrosis regression.^32^ It is clinically recognized that, around this stage, liver cirrhosis or Child‐Pugh grade B patients gradually lose the high regenerative ability.^33^ Thus, for maintaining or improving liver function, maintaining a liver stiffness <4000 Pa in MRE is a critical factor, and this can be achieved by educating patients about lifestyle and nutrition. We propose that, when the level of liver stiffness exceeds 4000 Pa, patients should be treated for the cause of the liver disease and simultaneously be provided supportive treatment for liver cirrhosis, such as BCAA, diuretics, synthetic disaccharides, and nonsystemic antibiotics. Furthermore, evaluation by MRE with cancer screening by gadolinium ethoxybenzyl‐diethylenetriaminepentaacetic acid (Gd‐EOB‐DTPA)‐enhanced MRI examination would be highly useful for specialists. Another alternative approach may be to offer yearly usage of cross‐sectional imaging methods for surveillance in low‐risk HCC patients detected using the noninvasive markers used in the present study. The application of an annual surveillance strategy with MRI has shown satisfactory performance in a recent study regardless of risk stratification.^34^ Therefore, reducing the intervals of surveillance imaging in low‐risk HCC patients may further increase the yield of surveillance and diminish cost and time concerns.

A limitation of this study was that data were collected from only one specialized institution that primarily treats patients with chronic liver disease. In addition, almost all of the high‐risk HCV and HBV patients were already being treated using nucleoside analogs or DAA. Future researchers should consider collecting data from multiple primary health facilities to confirm our results.

In conclusion, we showed that, using appropriate markers and tools, we can establish stepwise, practical, noninvasive, and etiology‐based screening for improving prognosis at the level of primary and specialty care depending on the situation of the facility. It is very important for doctors in clinics and specialists in hospitals to understand the available methods and share the proper role for following chronic liver disease depending on the etiology.

## Supporting information


**Appendix** **S1** Supplementary Information.

**Figure S1** Flow chart of this study. The highlighted areas indicate the factors to be considered by primary physicians (blue), factors correlated with MRE (red), and factors to be considered by specialists (green).

**Table S1** Characteristics of the patients with HBV, HCV, NASH, and ASH who underwent MRE.

## References

[1] Feld JJ , Jacobson IM , Hezode C *et al* Sofosbuvir and velpatasvir for HCV genotype 1, 2, 4, 5, and 6 infection. N. Engl. J. Med. 2015; 373: 2599–607. 26571066 10.1056/NEJMoa1512610

[2] Zeuzem S , Foster GR , Wang S *et al* Glecaprevir‐pibrentasvir for 8 or 12 weeks in HCV genotype 1 or 3 infection. N. Engl. J. Med. 2018; 378: 354–69. 29365309 10.1056/NEJMoa1702417

[3] Tang LSY , Covert E , Wilson E , Kottilil S . Chronic hepatitis B infection: a review. JAMA. 2018; 319: 1802–13. 29715359 10.1001/jama.2018.3795

[4] Tateishi R , Uchino K , Fujiwara N *et al* A nationwide survey on non‐B, non‐C hepatocellular carcinoma in Japan: 2011–2015 update. J. Gastroenterol. 2019; 54: 367–76. 30498904 10.1007/s00535-018-1532-5 PMC6437291

[5] Weinmann A , Alt Y , Koch S *et al* Treatment and survival of non‐alcoholic steatohepatitis associated hepatocellular carcinoma. BMC Cancer. 2015; 15: 210. 25884354 10.1186/s12885-015-1197-x PMC4407550

[6] Hassan I , Gane E . Improving survival in patients with hepatocellular carcinoma related to chronic hepatitis C and B but not in those related to non‐alcoholic steatohepatitis or alcoholic liver disease: a 20‐year experience from a national programme. Intern. Med. J. 2019; 49: 1405–11. 30908822 10.1111/imj.14304

[7] Vilar‐Gomez E , Calzadilla‐Bertot L , Wai‐Sun Wong V *et al* Fibrosis severity as a determinant of cause‐specific mortality in patients with advanced nonalcoholic fatty liver disease: a multi‐national cohort study. Gastroenterology. 2018; 155: 443–57.e17. 29733831 10.1053/j.gastro.2018.04.034

[8] Hagstrom H , Nasr P , Ekstedt M *et al* Fibrosis stage but not NASH predicts mortality and time to development of severe liver disease in biopsy‐proven NAFLD. J. Hepatol. 2017; 67: 1265–73. 28803953 10.1016/j.jhep.2017.07.027

[9] Angulo P , Kleiner DE , Dam‐Larsen S *et al* Liver fibrosis, but no other histologic features, is associated with long‐term outcomes of patients with nonalcoholic fatty liver disease. Gastroenterology. 2015; 149: 389–97.e10. 25935633 10.1053/j.gastro.2015.04.043 PMC4516664

[10] Yoneda M , Imajo K , Takahashi H *et al* Clinical strategy of diagnosing and following patients with nonalcoholic fatty liver disease based on invasive and noninvasive methods. J. Gastroenterol. 2018; 53: 181–96. 29177681 10.1007/s00535-017-1414-2 PMC5846871

[11] Chang Y , Cho YK , Kim Y *et al* Nonheavy drinking and worsening of noninvasive fibrosis markers in nonalcoholic fatty liver disease: a cohort study. Hepatology. 2019; 69: 64–75. 30019340 10.1002/hep.30170

[12] Demirtas CO , Gunduz F , Kani HT *et al* External validation of the Toronto hepatocellular carcinoma risk index in Turkish cirrhotic patients. Eur. J. Gastroenterol. Hepatol. 2020; 32: 882–8. 32395972 10.1097/MEG.0000000000001685

[13] Demirtas CO . Surveillance of hepatocellular carcinoma in cirrhotic patients: current knowledge and future directions. Hepatol. Forum. 2020. 10.14744/hf.2020.2020.0003 PMC9349494 35949723

[14] Toshima T , Shirabe K , Ikegami T *et al* A novel serum marker, glycosylated *Wisteria floribunda* agglutinin‐positive Mac‐2 binding protein (WFA(+)‐M2BP), for assessing liver fibrosis. J. Gastroenterol. 2015; 50: 76–84. 24603981 10.1007/s00535-014-0946-y

[15] Shirabe K , Bekki Y , Gantumur D *et al* Mac‐2 binding protein glycan isomer (M2BPGi) is a new serum biomarker for assessing liver fibrosis: more than a biomarker of liver fibrosis. J. Gastroenterol. 2018; 53: 819–26. 29318378 10.1007/s00535-017-1425-z

[16] Kaffe E , Katsifa A , Xylourgidis N *et al* Hepatocyte autotaxin expression promotes liver fibrosis and cancer. Hepatology. 2017; 65: 1369–83. 27981605 10.1002/hep.28973

[17] Tejima K , Masuzaki R , Ikeda H *et al* Thrombocytopenia is more severe in patients with advanced chronic hepatitis C than B with the same grade of liver stiffness and splenomegaly. J. Gastroenterol. 2010; 45: 876–84. 20339877 10.1007/s00535-010-0233-5

[18] Ito T , Ishigami M , Ishizu Y *et al* Utility and limitations of noninvasive fibrosis markers for predicting prognosis in biopsy‐proven Japanese non‐alcoholic fatty liver disease patients. J. Gastroenterol. Hepatol. 2019; 34: 207–14. 30144360 10.1111/jgh.14448

[19] Cassinotto C , Boursier J , de Ledinghen V *et al* Liver stiffness in nonalcoholic fatty liver disease: a comparison of supersonic shear imaging, FibroScan, and ARFI with liver biopsy. Hepatology. 2016; 63: 1817–27. 26659452 10.1002/hep.28394

[20] Osaki A , Kubota T , Suda T *et al* Shear wave velocity is a useful marker for managing nonalcoholic steatohepatitis. World J. Gastroenterol. 2010; 16: 2918–25. 20556839 10.3748/wjg.v16.i23.2918 PMC2887589

[21] Schwimmer JB , Behling C , Angeles JE *et al* Magnetic resonance elastography measured shear stiffness as a biomarker of fibrosis in pediatric nonalcoholic fatty liver disease. Hepatology. 2017; 66: 1474–85. 28493388 10.1002/hep.29241 PMC5650504

[22] Matsui N , Imajo K , Yoneda M *et al* Magnetic resonance elastography increases usefulness and safety of non‐invasive screening for esophageal varices. J. Gastroenterol. Hepatol. 2018; 33: 2022–8. 29869419 10.1111/jgh.14298

[23] Loomba R , Wolfson T , Ang B *et al* Magnetic resonance elastography predicts advanced fibrosis in patients with nonalcoholic fatty liver disease: a prospective study. Hepatology. 2014; 60: 1920–8. 25103310 10.1002/hep.27362 PMC4245360

[24] Imajo K , Kessoku T , Honda Y *et al* Magnetic resonance imaging more accurately classifies steatosis and fibrosis in patients with nonalcoholic fatty liver disease than transient elastography. Gastroenterology. 2016; 150: 626–37 e7. 26677985 10.1053/j.gastro.2015.11.048

[25] Wildman‐Tobriner B , Middleton MM , Moylan CA *et al* Association between magnetic resonance imaging‐proton density fat fraction and liver histology features in patients with nonalcoholic fatty liver disease or nonalcoholic steatohepatitis. Gastroenterology. 2018; 155: 1428–35 e2. 30031769 10.1053/j.gastro.2018.07.018 PMC6456892

[26] Mantovani A , Targher G . Type 2 diabetes mellitus and risk of hepatocellular carcinoma: spotlight on nonalcoholic fatty liver disease. Ann. Transl. Med. 2017; 5: 270. 28758096 10.21037/atm.2017.04.41 PMC5515814

[27] Younossi ZM . Non‐alcoholic fatty liver disease ‐ a global public health perspective. J. Hepatol. 2019; 70: 531–44. 30414863 10.1016/j.jhep.2018.10.033

[28] Kawai H , Nomoto M , Suda T *et al* Multicentric occurrence of hepatocellular carcinoma with nonalcoholic steatohepatitis. World J. Hepatol. 2011; 3: 15–23. 21307983 10.4254/wjh.v3.i1.15 PMC3035698

[29] Kaya E . The utility of noninvasive scores in non‐alcoholic fatty liver disease patients with normal and elevated serum transaminases. Hepatol. Forum. 2020; 1: 8–13. 10.14744/hf.2020.0006 PMC9344368 35949666

[30] Younossi ZM , Loomba R , Anstee QM *et al* Diagnostic modalities for nonalcoholic fatty liver disease, nonalcoholic steatohepatitis, and associated fibrosis. Hepatology. 2018; 68: 349–60. 29222917 10.1002/hep.29721 PMC6511364

[31] Abraldes JG , Bureau C , Stefanescu H *et al* Noninvasive tools and risk of clinically significant portal hypertension and varices in compensated cirrhosis: the “Anticipate” study. Hepatology. 2016; 64: 2173–84. 27639071 10.1002/hep.28824

[32] Nakazawa M , Imai Y , Uchiya H *et al* Balloon‐occluded retrograde transvenous obliteration as a procedure to improve liver function in patients with decompensated cirrhosis. JGH Open. 2017; 1: 127–33. 30483549 10.1002/jgh3.12020 PMC6207025

[33] Dezso K , Rokusz A , Bugyik E *et al* Human liver regeneration in advanced cirrhosis is organized by the portal tree. J. Hepatol. 2017; 66: 778–86. 27913222 10.1016/j.jhep.2016.11.014

[34] Demirtas CO , Gunduz F , Tuney D *et al* Annual contrast‐enhanced magnetic resonance imaging is highly effective in the surveillance of hepatocellular carcinoma among cirrhotic patients. Eur. J. Gastroenterol. Hepatol. 2020; 32: 517–23. 31524775 10.1097/MEG.0000000000001528

