# Supplementary material for: Screening and follow‐up of chronic liver diseases with understanding their etiology in clinics and hospitals
Source: JGH Open. 2020 Aug 24;4(5):827–37. doi: 10.1002/jgh3.12406 (PMC7578295; doi:10.1002/jgh3.12406)
Supplement: Supplementary file 1 — Appendix S1 Supplementary Information. [file JGH3-4-827-s001.docx]

**Supplemental Materials and Methods**

**Serum markers of fibrosis**

On the same day or soon after the MRE examination, 7 ml of blood specimens was obtained. Serum M2BPGi (Sysmex, Hyogo, Japan) and ATX (Tosoh, Tokyo, Japan) levels were measured based on a sandwich immunoassay using the fully automatic immunoanalyzer HISCL-system (Sysmex) and AIA-system (Tosoh), respectively. Serum aminotransferase (AST), aminotransferase (ALT), and platelet counts were measured in the same laboratory. FIB-4 index was calculated using the equation [(Age × AST)/(PLT × (ALT)1/2)].

**Imaging assessments of liver stiffness**

USE was performed using an ACUSON S2000 ultrasound system (SIEMENS Medical Solutions Inc., Malvern, PA, USA). A single ultrasonic transducer array was used to apply short, localized radiation forces within the tissue and to track the resulting tissue dynamic response with time. During a regular ultrasound observation, a rectangular region-of-interest (ROI) was set and acoustic pulses were radiated to displace the tissues downward, beside the ROI. Once the tissues are displaced, the shear stress restores the tissue to its previous position and generates shear waves, which travel perpendicular to the direction of the acoustic push pulse. The machine continuously monitors tissue positions in the ROI and automatically calculates the speed of the waves in m/s.

MRE was performed using a 3.0 T clinical whole-body MR scanner (Discovery 750W, GE, Milwaukee, WI, USA) along with a 32-element phased-array coil. A passive driver was positioned on the right rib cage at the level of the xiphoid process and attached to a waveform generator. Four slices of MRE were obtained including the level of porta hepatis under 24 s of breath holding. The parameters of 2D spin-echo echo-plannar-MRE sequence were as follows: TE, 59.2 ms; TR, 1000 ms; slice thickness, 8.0 mm; intersection gap, 4.0 mm; field-of-view, 42 × 42 cm; matrix, 64 × 64; scan plane, axial; magnetization encoding gradients frequency, 80 Hz; driver frequency, 60 Hz; driver amplitude, 60 Hz. Wave images and stiffness map with crosshatching were automatically generated on the operating console. Liver stiffness was measured using stiffness map with crosshatching by an experienced abdominal radiologist, who was blinded to the clinical data. The ROI was set in the right lobe to avoid the effect of heartbeat and more than 1 cm inside from the liver edge to exclude the edge effect.

Collateral circulation was evaluated by esophagogastroduodenoscopy (EGD) and computed tomography (CT) performed before evaluation of liver fibrosis. To evaluate varices, the previously reported classification was used for the form (F) of the varices: F1, straight and small; F2, moderately enlarged and beady; and F3, markedly enlarged and nodular; patients with more than F1 varices were evaluated to be positive for collateral circulation. Evaluation using computed tomography was checked by collateral development at the watershed zones between the portal venous drainage (stomach, small bowel, large bowel, and spleen) and systemic venous drainage (esophagus, distal rectum, retroperitoneal veins) or by collateral development from the recanalization of embryological connections (e.g., umbilical vein, persistent ductus venosus) between the portal venous tributaries and systemic veins. If these collateral veins were detected, the patients were evaluated to be positive for collateral circulation.

**Liver histology**

Histological sections of the liver tissues obtained by needle biopsy or surgery due to hepatocellular carcinoma were analyzed. For the chronically damaged livers, we scored the fibrosis stages as: stage 0 (0), no scarring; stage 1 (F1), minimal scarring; stage 2 (F2), scarring has occurred and extends outside the areas in the liver that contain blood vessels; stage 3 (F3), bridging fibrosis is spreading and connecting to other areas that contain fibrosis; and stage 4 (F4), cirrhosis. Histological activity was scored according to the METAVIR algorithm. The tissue fibrosis stages were compared with the results of MRE and USE.

**Diagnosis of HCC**

In typical cases, HCC was diagnosed based on the elevated levels of tumor markers such as alpha-fetoprotein (AFP), lens culinaris-agglutinin-reactive fraction of AFP (AFP-L3), des-γ-carboxy prothrombin (DCP), dynamic CT, or gadolinium-ethoxybenzyl-diethylenetriamine pentaacetic acid (Gd-EOB-DTPA)-enhanced MRI. A liver biopsy was performed to diagnose HCC in atypical cases.

**Statistical analysis**

The associations among MRE, USE, and serum markers were determined using Mann–Whitney U test and spearman correlations. The discrimination of imaging results and serum markers for the diagnostic efficiency for HCC and collaterals and for assessing liver fibrosis (F0-2 versus F3-4, F0-3 versus F4) were evaluated using the Mann-Whitney U test and area-under-the-receiver operating characteristic curve (AUROC). Diagnostic cut-offs for predicting the diagnosis of HCC and collaterals and for assessing liver fibrosis were determined based on the maximal sum of sensitivity and specificity. Area under the curve (AUC) was compared using the z test. All statistical analyses were performed using Prism 6 for Windows (ver. 6.07) (La Jolla, CA, USA) and a two-sided P-value of less than 0.05 was considered statistically significant.
