# Supplementary figures and images for: Screening and follow‐up of chronic liver diseases with understanding their etiology in clinics and hospitals
Source: JGH Open. 2020 Aug 24;4(5):827–37. doi: 10.1002/jgh3.12406 (PMC7578295; doi:10.1002/jgh3.12406)

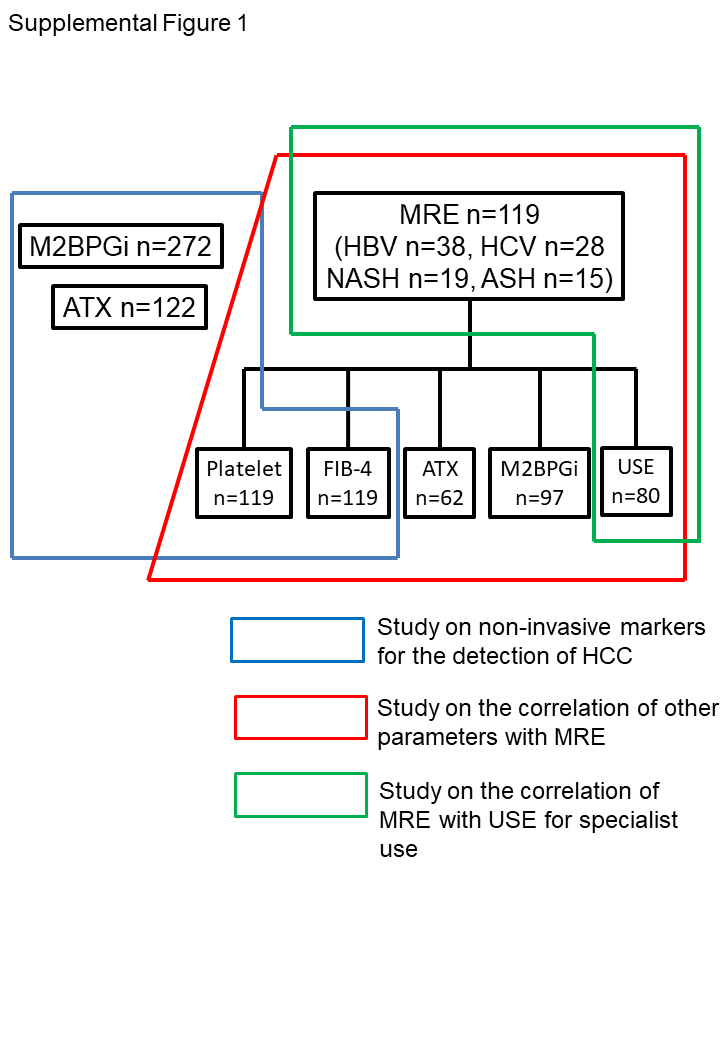

Supplement: Supplementary file 2 — Figure S1 Flow chart of this study. The highlighted areas indicate the factors to be considered by primary physicians (blue), factors correlated with MRE (red), and factors to be considered by specialists (green). [file JGH3-4-827-s002.TIF]

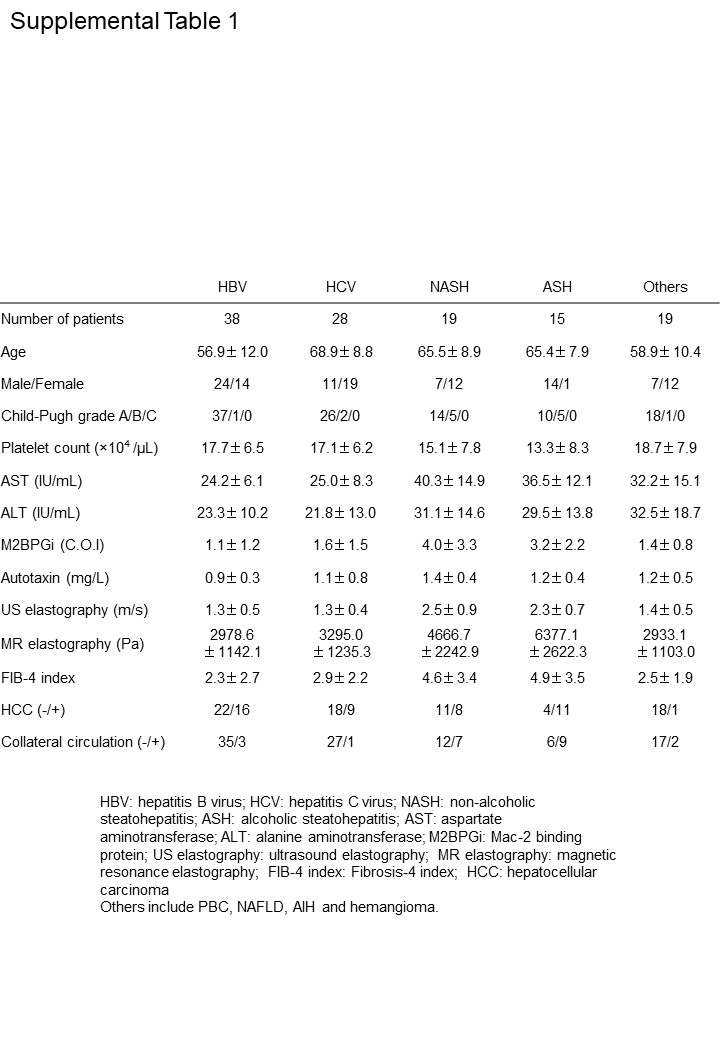

Supplement: Supplementary file 3 — Table S1 Characteristics of the patients with HBV, HCV, NASH, and ASH who underwent MRE. [file JGH3-4-827-s003.TIF]
